# The prelude to industrial whaling: identifying the targets of ancient European whaling using zooarchaeology and collagen mass-peptide fingerprinting

**DOI:** 10.1098/rsos.230741

**Published:** 2023-09-13

**Authors:** Youri van den Hurk, Fanny Sikström, Luc Amkreutz, Madeleine Bleasdale, Aurélia Borvon, Brice Ephrem, Carlos Fernández-Rodríguez, Hannah M. B. Gibbs, Leif Jonsson, Alexander Lehouck, Jose Martínez Cedeira, Stefan Meng, Rui Monge, Marta Moreno, Mariana Nabais, Carlos Nores, José Antonio Pis-Millán, Ian Riddler, Ulrich Schmölcke, Martin Segschneider, Camilla Speller, Maria Vretemark, Stephen Wickler, Matthew Collins, Marie-Josée Nadeau, James H. Barrett

**Affiliations:** ^1^ Department of Archaeology and Cultural History, University Museum, Norwegian University of Science and Technology, Trondheim, Norway; ^2^ National Laboratory for Age Determination, NTNU Vitenskapsmuseet, Norwegian University of Science and Technology, Erling Skakkes Gate 47b, 7491 Trondheim, Norway; ^3^ Groningen Institute of Archaeology, University of Groningen, Groningen, The Netherlands; ^4^ National Museum of Antiquities, Papengracht 30, 2301EC Leiden, The Netherlands; ^5^ Faculty of Archaeology, Leiden University, Einsteinweg 2, 2333 Leiden, The Netherlands; ^6^ Department of Archaeology, University of York, York, UK; ^7^ CNRS, UMR 7041 ArScAn Equipe Archéologies Environnementales, Nanterre, France; ^8^ Laboratoire d'Anatomie Comparée, ONIRIS (École Nationale Vétérinaire, Agroalimentaire et de l'Alimentation, Nantes-Atlantique), Nantes, France; ^9^ CNRS, UMR 6566 CReAAH Laboratoire Archéosciences, University of Rennes, Campus de Beaulieu, 35042 Rennes Cedex, France; ^10^ Department of History, Universidad de León, León, Spain; ^11^ Institute of Archaeology, University College London, London, UK; ^12^ Osteology, Aschebergsgatan 32, Gothenburg, SE 41133, Sweden; ^13^ Abbey Museum of the Dunes, Koninklijke Prinslaan 6–8, 8670 Koksijde, Belgium; ^14^ Coordinadora para o Estudio dos Mamíferos Mariños, Rúa do Ceán, No 2, 36350 Nigrán, Pontevedra, Spain; ^15^ Institute of Geography and Geology, University of Greifswald, Friedrich-Ludwig-Jahn Strasse 17A, 17487 Greifswald, Germany; ^16^ UNIARQ – Centro de Arqueologia da Universidade de Lisboa, Faculdade de Letras, Universidade de Lisboa, Lisbon, 1600-214, Portugal; ^17^ Instituto de Historia - CSIC, Albasanz 26-28, Madrid, 28037, Spain; ^18^ IPHES-CERCA - Institut Català de Paleoecologia Humana i Evolució Social, Zona Educacional 4, Campus Sescelades URV (Edifici W3), 43007 Tarragona, Spain; ^19^ Departament d'Història i Història de l'Art, Universitat Rovira i Virgili, Avinguda de Catalunya 35, 43002 Tarragona, Spain; ^20^ INDUROT – Instituto de Recursos Naturales y Ordenación del Territorio, Universidad de Oviedo, Mieres, 33600, Spain; ^21^ Centro de Experimentación Pesquera, Dirección General de Pesca Marítima, Gobierno del Principado de Asturias, 33212 Gijón, Spain; ^22^ Independent Researcher, Schloss Gottorf, Schleswig, Germany; ^23^ Centre for Baltic and Scandinavian Archaeology (ZBSA), Schloss Gottorf, Schleswig, Germany; ^24^ Lower Saxony Institute for Historical Coastal Research, Viktoriastrasse 26/28, 26382 Wilhelmshaven, Germany; ^25^ Department of Anthropology, University of British Columbia, 6303 NW Marine Drive, Vancouver V6T 1Z1, Canada; ^26^ Västergötlands Museum, Skara, Sweden; ^27^ The Arctic University Museum of Norway, 9037 Tromsø, Norway; ^28^ Department of Archaeology, University of Cambridge, Cambridge, UK; ^29^ The Globe Institute, University of Copenhagen, Kobenhavns, Denmark

**Keywords:** zooarchaeology, historical ecology, whales, zooarchaeology by mass spectrometry

## Abstract

Taxonomic identification of whale bones found during archaeological excavations is problematic due to their typically fragmented state. This difficulty limits understanding of both the past spatio-temporal distributions of whale populations and of possible early whaling activities. To overcome this challenge, we performed zooarchaeology by mass spectrometry on an unprecedented 719 archaeological and palaeontological specimens of probable whale bone from Atlantic European contexts, predominantly dating from *ca* 3500 BCE to the eighteenth century CE. The results show high numbers of Balaenidae (many probably North Atlantic right whale (*Eubalaena glacialis*)) and grey whale (*Eschrichtius robustus*) specimens, two taxa no longer present in the eastern North Atlantic. This discovery matches expectations regarding the past utilization of North Atlantic right whales, but was unanticipated for grey whales, which have hitherto rarely been identified in the European zooarchaeological record. Many of these specimens derive from contexts associated with mediaeval cultures frequently linked to whaling: the Basques, northern Spaniards, Normans, Flemish, Frisians, Anglo-Saxons and Scandinavians. This association raises the likelihood that early whaling impacted these taxa, contributing to their extirpation and extinction. Much lower numbers of other large cetacean taxa were identified, suggesting that what are now the most depleted whales were once those most frequently used.

## 1. Introduction

Whaling has been undertaken in European waters for millennia. This activity, in particular commercial whaling, has completely altered the composition of whale populations and by extension transformed ecosystems [1]. The impact of industrial whaling in the twentieth century is particularly well documented, including reductions in both numbers and size for many large whale species [2,3]. Numerous conservation efforts now attempt to protect whales [4–7]. However, despite recent advances in the study of archaeological whale remains (e.g. [8,9]), we still lack deep-time baseline empirical data concerning the spatio-temporal range and human exploitation of past populations. In order to manage whale species more effectively, an improved understanding of past populations and past whaling activities has to be achieved. By reconstructing past ranges and migration patterns, and facilitating inferences regarding changes in demography, data from historical ecology can be used to inform the protection of potential migration routes and foraging grounds. This observation is made all the more pertinent by the recent movement of a few Pacific grey whales (*Eschrichtius robustus*) into the Atlantic, presumably via the Northwest Passage in the context of climate change [10] on the one hand, and by the increasingly precarious status of the small population of remaining North Atlantic right whales (*Eubalaena glacialis*) on the other [11].

The beginnings of whaling activities in Europe are hard to pinpoint with clarity. Both the use of stranded individuals and active hunting in prehistory have long been recognized [12], with finds of whale barnacles suggesting the utilization of whales may already have been practised in the Pleistocene [8,13]. Mediaeval cultures are especially associated with the early growth of whaling, but the targets and scale of these activities are poorly understood given the limitations of pre-modern historical records [14]. Our aim is to ascertain which whale taxa were most used in the period prior to industrial whaling, and in particular how the frequencies of Atlantic grey whale and Balaenidae (including North Atlantic right whales) compare with other taxa. Throughout this paper, whaling means actively pursuing and hunting whales, opportunistic scavenging is making use of stranded whales (either dead or alive), and utilization or exploitation means either of these two alternatives.

Subfossil whale bone specimens from archaeological and palaeontological contexts hold an important key to revealing the past. However, whale bones are infamously hard to identify to the family or species level. This difficulty can be ascribed to limited osteological morphological differences between the taxa, the often fragmented state of the remains, and the lack of extensive reference collections [15]. As a result, many subfossil whale bones have merely been identified as ‘whale’.

Zooarchaeology by mass spectrometry (ZooMS) has been shown to be an excellent method for identifying whale bone specimens to family or species [8]. Relevant European-focused ZooMS case studies exist regarding Scotland [16–19], Norway [20], France [15,21], Spain [10,22], Iceland [16], Morocco [10], Italy [15], the Low Countries [23] and Scandinavia [24]. Here, we greatly expand on this work by contributing 719 new ZooMS analyses of subfossil specimens that cross the spatial and/or chronological boundaries of previous research.

This paper asks which whales were most frequently used by past coastal societies in Europe, from Iberia to northern Norway. We focus predominantly on subfossil whale bone specimens of Iron Age to mediaeval date (broadly *ca* 900 BCE to *ca* 1500 CE), with a few earlier and later specimens providing a broader chronological context. We acknowledge that not all archaeological specimens derive from actively caught individuals, but by studying whale bone remains from sites from cultures frequently associated with whaling, inferences can potentially be made regarding past whaling activities. We address this question by determining the taxa of archaeological finds of cetacean bone using ZooMS. High chronological precision is not a present goal, being a matter of ongoing research given the complexity of radiocarbon dating migratory marine taxa, some of which feed in waters above the northern latitudinal limit of the Marine20 calibration curve (see [25]).

In result, using published historical evidence as background information, we infer that now extinct or critically endangered cetaceans—the Atlantic grey whale and the North Atlantic right whale (here represented by specimens identified to the family Balaenidae)—were especially frequently targeted by human hunters prior to being overexploited. These two taxa were particularly vulnerable to early whaling activities as they were slow swimmers with near-coastal habitats. This interpretation matches expectations regarding North Atlantic right whales, but is an unanticipated discovery for Atlantic grey whales, which are very poorly documented in historical texts and have hitherto rarely been identified in the European zooarchaeological record. In comparative perspective, it is relevant that seemingly superabundant taxa, for reasons of accessibility to potential human harvesters, can be counterintuitively vulnerable to overexploitation or extinction. Classic cases of this phenomenon are known from both the recent and distant past, among both marine and terrestrial/avian fauna. Examples include the collapse of Newfoundland's northern cod (*Gadus morhua*) in 1992/1993 [26] and the European sea sturgeon (*Acipenser sturio*) in the early twentieth century [27], as well as the extinction of the great auk (*Pinguinus impennis*) in the mid-nineteenth century [28] and the passenger pigeon (*Ectopistes migratorius*) in the early twentieth century [29]. This phenomenon can be understood as an ecological dimension of the so-called resource curse, where (for complex cultural and ecological reasons) socio-environmental systems with initially abundant natural resources may not ultimately thrive [30,31]. Concurrently, this study also sheds important new light on the exploitation (by active whaling and/or the use of strandings) of other cetacean taxa. Its findings are of direct importance to marine historical ecology and of wider relevance to our understanding of the socioecological processes that can lead to the overexploitation of seemingly abundant taxa.

## 2. Material and methods

**Figure 1.**
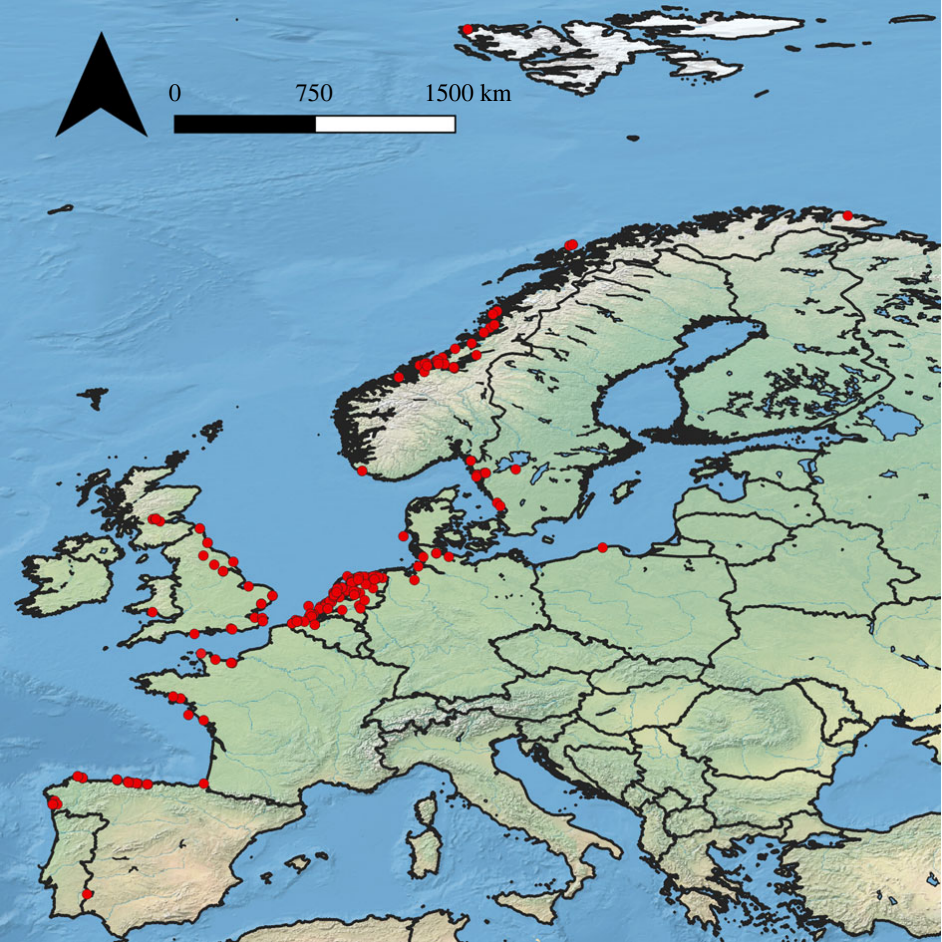
Locations of the 719 probable whale bone specimens analysed as part of this study (basemap after [32]).

To assess the past geographical range of whale taxa in eastern North Atlantic waters, and to reconstruct which taxa were most exploited, a total of 719 subfossil probable cetacean specimens were collected from northern and western Europe (figure 1). The sampling aimed to maximize spatial coverage along the seaboard of Atlantic Europe, from Iberia to northern Norway, as this is the suspected former range of both the North Atlantic right whale and the grey whale [10]. The majority of the specimens derived from archaeological contexts (electronic supplementary material, table S1); several palaeontological finds were also included. A particular focus was placed on several spatio-temporal zones of cultures associated with early whaling activities. These include the mediaeval cultures of the Basques, northern Spaniards, Normans, Flemish, Frisians, Anglo-Saxons and Scandinavians [14,23,33–36]. Some earlier specimens were also considered for comparison—in particular, material from sites in The Netherlands of the maritime-oriented Middle to Late Neolithic Vlaardingen Culture (*ca* 3500–2500 BCE) [37]. Specimens likely to derive from a large whale species were prioritized. The practicalities of availability (as is typical for archaeological and palaeontological research) meant that several localities produced a large number of specimens, while many others provided few. When multiple specimens derived from the same context from the same site, only one specimen was sampled in order to minimize the risk of sampling the same whale individual multiple times. Samples were taken by the authors, but were also contributed by numerous local collaborators, allowing a large geographical area to be considered. Samples were collected from April 2021 to June 2022.

For ZooMS analysis of these 719 specimens, samples were taken using a Dremel® rotary tool removing a small piece of bone weighing up to *ca* 500 mg. Each rotary disc was used once, while the tool was cleaned using ethanol in between sampling. For 474 specimens, collagen was extracted using a modified Longin [38] method as detailed in Seiler *et al*. [39], with the addition of a lipid extraction step and the use of a higher acid concentration, at the National Laboratory for Age Determination, Norwegian University of Science and Technology, Norway.

Initially, the samples were crushed to small pieces, placed into test-tubes, and cleaned in an ultrasonic bath with 18.2 MΩ cm ultrapure water (type 1) (three times 5 min). The samples were then ultrasonicated for 15 min in dichloromethane and methanol (2 : 1). This step was repeated three or more times, until the solution was clear. Following this, the material was demineralized overnight using 2.44 M HCl (50 ml of solution per 100 mg of bone) in glass tubes within a vacuum desiccator kept at room temperature. The HCl was removed from the samples and *ca* 8 ml of ultrapure water was repeatedly added to the tube and removed until a pH of 3–4 was reached. Subsequently, 4 ml of 0.5% NaOH was added for 2–4 h at room temperature to dissolve humic acids. The NaOH was removed from the samples and *ca* 8 ml of ultrapure water was repeatedly added to the tube and removed until pH < 10. Afterwards, 5 ml of 1.22 M HCl was added for 1–2 h to remove atmospheric CO_2_. The sample was then washed repeatedly with ultrapure water until pH = 3 ± 0.2 and hydrolysed to gelatin at 70°C overnight. Finally, the gelatin was filtered through a prebaked (900°C) quartz filter (Merck Millipore, AQFA04700, 99.998% capture for 0.3 µm particles) and the filtrate freeze dried. The final collagen was taken to the Henry Wellcome Laboratory for Biomolecular Archaeology, University of Cambridge, UK, for ZooMS analysis.

Collagen extraction failed for 25 specimens and when sufficient original bone material remained a subsample was taken directly to the Henry Wellcome Laboratory. Additionally, another 237 specimens were analysed by ZooMS without prior collagen extraction. For these 262 (25 + 237) specimens, approximately 30 mg subsamples were taken and processed at the Henry Wellcome Laboratory for Biomolecular Archaeology. The bones were demineralized in 0.6 M hydrochloric acid at 4°C for two weeks. Each sample was then centrifuged, the hydrochloric acid discarded, and the retained material rinsed three times with 200 µl of 50 mMol ammonium bicarbonate (AmBic) pH 8.0 solution, before being gelatinized in 100 µl of AmBic solution at 65°C for 1 h. For the 449 collagen samples prepared at the Norwegian University of Science and Technology (NTNU) (474 samples, minus the 25 which failed), 0.1 mg of collagen was also placed in AmBic solution at 65°C for 1 h.

For both the 262 and 449 sample groups, the gelatinized collagen, still in AmBic, was incubated with 0.4 µg of trypsin at 37°C overnight, and subsequently acidified with 0.1% trifluoroacetic acid (TFA). The collagen was then purified using a 100 µl C18 resin ZipTip pipette tip (EMD Millipore) with conditioning and eluting solutions composed of 50% acetonitrile and 0.1% TFA and washing solution composed of 0.1% TFA; the samples were eluted in a volume of 50 µl. Equal amounts of the collagen extract and α-cyano-hydroxycinnamic acid matrix solution (1% in conditioning solution) were mixed (1 µl each) and spotted onto a 384 spot MALDI target plate. Each sample was externally calibrated against an adjacent spot containing a mixture of six peptides (des-Arg1-bradykinin *m/z* = 904.681, angiotensin I *m/z* = 1295.685, Glu1-fibrinopeptide B *m/z* = 1750.677, ACTH (1–17 clip) *m/z* = 2093.086, ACTH (18–39 clip) *m/z* = 2465.198 and ACTH (7–38 clip) *m/z* = 3657.929). Samples were spotted in triplicate, and run on a Bruker ultraflex III MALDI tandem time-of-flight (TOF/TOF) mass spectrometer with a Nd:YAG smart beam laser. A SNAP averaging algorithm was used to obtain monoisotopic masses (C 4.9384, N 1.3577, O 1.4773, S 0.0417, H 7.7583). Averaged spectra were created from the replicates for each specimen using mMass software [40], and then visually compared with published *m/z* markers for mammals, as presented in Buckley *et al*. [41], Kirby *et al*. [42], Buckley *et al*. [16] and Hufthammer *et al*. [20]. An additional eight specimens were previously analysed at BioArch at the University of York following the protocol outlined in Rodrigues *et al*. [10] and the results incorporated into this study.

While ZooMS reference spectra have been created for many large whale species, this has not been undertaken for all. Taxa without existing spectra include the Bryde's whale (*Balaenoptera edeni brydei*), Eden's whale (*Balaenoptera edeni edeni*), Omura's whale (*Balaenoptera omurai*), Rice's whale (*Balaenoptera ricei*), Antarctic minke whale (*Balaenoptera bonaerensis*), pygmy right whale (*Caperea marginata*), North Pacific right whale (*Eubalaena japonica*) and southern right whale (*Eubalaena australis*). Their omission prohibits identification of these taxa and could lead to misidentifications. However, the relevant missing control spectra are of taxa that are unlikely to have been hunted in the North Atlantic within the timeframe this study is concerned with.

For the taxa of central present concern, *E. robustus* can typically be identified to species using ZooMS, but *E. glacialis* cannot. The latter is instead classified as Balaenidae, together with the southern right whale (*E. australis*), the North Pacific right whale (*E. japonica*) and the bowhead whale (*Balaena mysticetus*). Of these, only the North Atlantic right whale and the bowhead whale are probable in a North Atlantic context. Differentiation between the North Atlantic right whale and the bowhead whale is difficult using ZooMS or osteological morphological identification. Moreover, the distribution of these taxa may have overlapped more substantially in the past than present. This observation is underlined by McLeod *et al*. [43], who using aDNA analysis, showed that the bowhead whale and not the North Atlantic right whale was the main target of Basque whalers in the western North Atlantic as was previously assumed. Though future aDNA analysis is needed to confirm the final identification of Balaenidae specimens, based on the spatio-temporal coverage of this study, largely using samples from northern and western Europe from *ca* 900 BCE to *ca* 1500 CE, *E. glacialis* is most likely for Balaenidae identifications. We base this inference not only on the range of the bowhead whale, which is restricted to Arctic and subarctic waters, but (most crucially) its tendency to stay close to the edge of the sea ice [44]. During the Holocene period (*ca* 11.7 ka BP to present), sea ice did not reach most of northern or western Europe [45].

Therefore, we suggest, with some caution, that the vast majority of the identified Balaenidae will probably be North Atlantic right whales, except for the northernmost specimens (e.g. from Svalbard (two specimens) and mainland Norway) and a few bones (e.g. from The Netherlands (11 specimens)) that may represent trophies from long-range whaling voyages of the post-mediaeval period. From the early seventeenth century, European whalers ventured towards the Arctic to hunt the bowhead whale and occasionally brought back bones. Often the mandibles were transported, of which 11 have been identified based on morphology after comparison with Turner [46] and modern reference material (see electronic supplementary material, table S1).

## 3. Results

**Figure 2.**
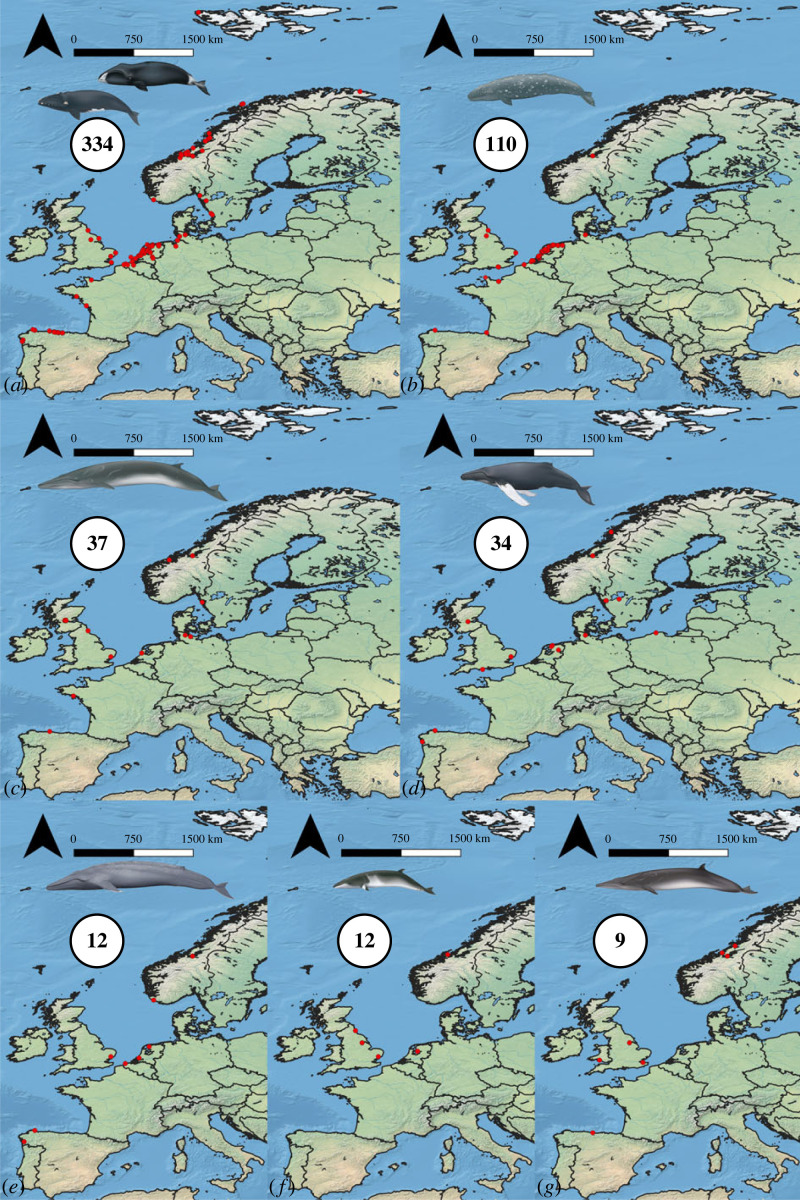
Spatial distribution of identified large whale specimens. (a) Balaenidae, (*b*) grey whale, (*c*) fin whale, (*d*) humpback whale, (*e*) blue whale, (*f*) common minke whale, (*g*) sei whale (whale illustrations © Uko Gorter, reproduced with permission). Numbers in maps are numbers of identified specimens (NISP) (basemap after [32]).

Using ZooMS, it proved possible to identify 623 specimens to cetacean taxon/ZooMS group level (figure 2, electronic supplementary material, figure S1 and table S1). For examples of ZooMS spectra for each cetacean group see electronic supplementary material, file 1. This is the largest and most spatially expansive dataset of identified subfossil whale specimens known to the authors. For another 26 specimens, ZooMS identification to a larger group of cetaceans was possible. Thirty-one specimens were identified as not belonging to a cetacean taxon, instead representing animals whose fragmentary bones could be visually mistaken for whale: e.g. cattle/aurochs (*Bos taurus/primigenius*), elephant/mammoth (Elephantidae), seal (Phocidae), walrus (*Odobenus rosmarus*) and even human (*Homo sapiens*). No results were reached for 39 specimens.

**Figure 3.**
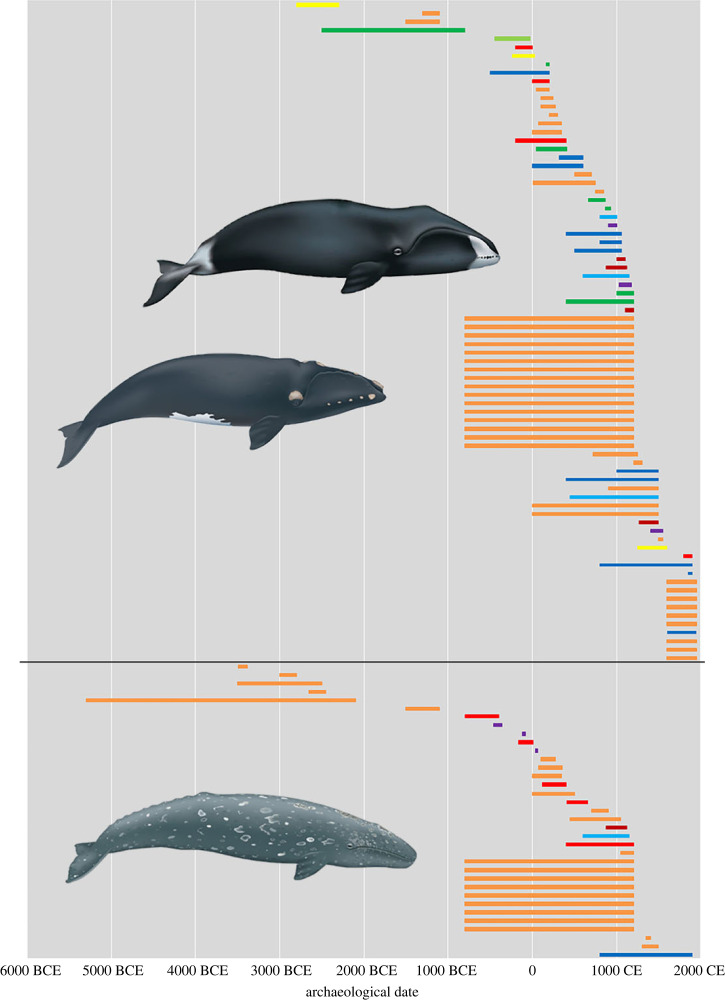
Temporal overview of identified Balaenidae (North Atlantic right whale/Bowhead whale) and grey whale specimens from contexts with archaeological dates. Country colour codes: Sweden = yellow, The Netherlands = orange, England = dark green, Portugal = light green, Spain = red, Norway = dark blue, Germany = light blue, Belgium = dark red, France = purple (whale illustrations © Uko Gorter, reproduced with permission).

By far the best represented group are the Balaenidae (334 specimens/46.5%) and the grey whale (110 specimens/15.3%). Specimens of both groups were found along the coast of Atlantic Europe, from Iberia to Norway, although the majority of the grey whale samples originate from The Netherlands (figures 2 and 3). The results more than double the number of Balaenidae specimens previously identified using ZooMS in European contexts. They also more than double the number of known grey whale specimens from the eastern North Atlantic (identified either through ZooMS or aDNA analysis), providing a new basis from which to assess the extirpation of the species ([47]; electronic supplementary material, table S2).

Additionally, some specimens of the sperm whale (*Physeter macrocephalus*) (43 specimens/6.0%), fin whale (*Balaenoptera physalus*) (37 specimens/5.1%) and humpback whale (*Megaptera novaeangliae*) (34 specimens/4.7%) were identified. Lower numbers were observed for the blue whale (*Balaenoptera musculus*), common minke whale (*Balaenoptera acutorostrata*) and sei whale (*Balaenoptera borealis*).

Besides sperm whale, the ZooMS analysis identified several other Odontoceti, often to groups of species that have similar peptide mass spectra (electronic supplementary material, figure S1 and table S2). These identifications broaden our knowledge of which whales were used in the past. However, they are not as informative as the great whale evidence in terms of the relative frequency of utilization because bones recognizably from small whales were not targeted for sampling. Aside from sperm whale, the identified Odontoceti specimens were highly fragmented pieces of ‘whale bone’ that did not allow *a priori* inferences regarding taxa or size based on morphology.

## 4. Discussion

### 4.1. Relative abundance and chronology by taxon

Taxa that were the main foci of twentieth-century whaling (e.g. blue, fin, sei, humpback and sperm whale [48]), and/or that are most abundant today in European waters (e.g. common minke whale [49]), are rarely represented among the archaeological and palaeontological finds considered in this study. Conversely, taxa that are either extinct (Atlantic grey whale) or no longer found in the area (family Balaenidae, with most inferred to be North Atlantic right whale) are the two most frequent archaeological finds. This counterintuitive observation supports the hypothesis that now-extinct or critically endangered cetaceans were especially frequently targeted by human hunters in the distant past. That right whales were frequently targeted by pre-industrial whalers is consistent with historical evidence [50], but the frequency of grey whale identifications is surprising in light of both the few previous subfossil finds and previous arguments that the population was small and unviable irrespective of human harvesting [51]. Note that some Balaenidae whales were still caught in the North Atlantic during the twentieth century, but in considerably lower numbers than the rorquals and the sperm whale [48,52].

By far the highest proportion of specimens has been identified as Balaenidae. While it cannot be excluded that some of these derive from bowhead whales (at least 11 mandibles and one atlas certainly do based on morphology), it is likely, based on the reasoning described above, that many or most represent the North Atlantic right whale. At least a millennium of hunting along the European coastline by various cultures frequently associated with whaling (e.g. Basques, northern Spaniards, Normans, Flemish, etc.) has led to the complete extirpation of what must once have been an at-least-coastally abundant population [33,35,47]. The temporal distribution of the specimens, based on archaeological context in almost all cases, shows high numbers in the Roman Period/Roman Iron Age (*ca* 100 BCE to *ca* 400 CE) and subsequent Middle Ages (*ca* 400 to *ca* 1500 CE). These were centuries of known or hypothesized pre-industrial whaling [10,23,34,53].

Though ZooMS does not provide data regarding the population size of the different taxa, the high number of Balaenidae, which are assumed, based on previous described reasons, to largely represent North Atlantic right whales, and their spatial distribution, suggests the species was widespread in European coastal waters until at least the Middle Ages. Their representation in the archaeological record implies that they were frequently used. The species has a near-coastal habitat, is a slow swimmer and is naturally buoyant after death ([54], 28–30). It was thus a comparatively easy target—the ‘right’ whale to hunt—for the multiple whaling societies that once existed along its migration paths in European waters. It may also have frequently stranded, providing a supplement to hunted individuals [44,47].

The high number of grey whale specimens strongly contrasts with the previously known subfossil record [51] and implies that the species was more frequently used in European waters than assumed hitherto. Like the North Atlantic right whale, the grey whale has a nearshore habitat and migratory behaviour. Thus, its availability to pre-industrial whalers (in addition to strandings) may also have contributed to the high representation of this species in comparison with other more pelagic taxa. Grey whale specimens are especially common in sites adjacent to the shallow waters of the southern North Sea, suggesting this area, rich in benthic biodiversity [55], might have been an important habitat and foraging ground. The grey whale specimens are predominately of Roman/Roman Iron Age to mediaeval date, from *ca* 100 BCE to *ca* 1200 CE. Neolithic specimens from sites of the Vlaardingen Culture demonstrate that grey whales were also used earlier (see below). No closely dated specimens in this dataset are later than the fifteenth century CE, consistent with extinction of the Atlantic grey whale in the middle centuries of the second millennium CE [51].

The fact that the Balaenidae (likely North Atlantic right whale) and grey whale, the two species that are currently no longer present in the eastern North Atlantic, are by far the highest represented species, indicates how significantly whale stocks have been altered in European waters after centuries of whaling activities. Alter *et al*. [51] previously argued that the genetic diversity of the grey whale in the North Atlantic declined over an extended period of time, pre-dating the onset of intensive whaling. They suggested that the decline of the species in the North Atlantic might have been precipitated by Holocene climate or other ecological causes. The present discovery of many archaeological specimens of grey whale, mostly dating between *ca* 100 BCE and *ca* 1200 CE (figure 3), instead raises the possibility that a critical blow to at least the eastern North Atlantic population was delivered by mediaeval whalers.

Other whale taxa are notable for their comparative scarcity in the subfossil record, but there is a cluster of sperm whale finds from the southern North Sea area. Unlike the grey whale, these specimens probably do not indicate that the species was especially abundant in the region. The southern North Sea has been described as a sperm whale trap [56]. Young bulls accidentally enter the northern North Sea and subsequently swim towards the southern coastline where the coastal configuration is characterized by expanses of sandbanks, estuaries and mudflats which interfere with their echolocation. This leads to strandings [56]. Vanselow *et al*. [57] also suggest that solar flares contribute to sperm whale strandings in the southern North Sea as a result of fluctuations in the Earth's geomagnetic field. Smeenk [56] provides a spatio-temporal overview of sperm whale strandings in the North Sea area, which reflects the distribution of the identified subfossil finds of this species.

The particularly scarce evidence of common minke whales is notable. Based on aerial surveys, minke whales appear to be the most common baleen whale species in the North Sea area [49]. The low representation of common minke whales might be the result of the species not being heavily targeted by early whalers, unlike the other large baleen whale taxa [2]. As a result, this species is less represented in the archaeological record and was also less historically depleted, resulting in a relatively high common minke whale population in modern times.

Beyond new evidence regarding the extirpated and extinct right and grey whale, the distribution of the subfossil finds by taxon is broadly consistent with what is known based on modern data. A specimen of beluga (*Delphinapterus leucas*; an Arctic taxon) from Uddevalla, Sweden, is an outlier, but it is undated and of uncertain significance. Belugas occurred in more southern waters in colder periods in the past [58], and also occasionally stray outside their normal range [59].

### 4.2. Inferring whaling

**Figure 4.**
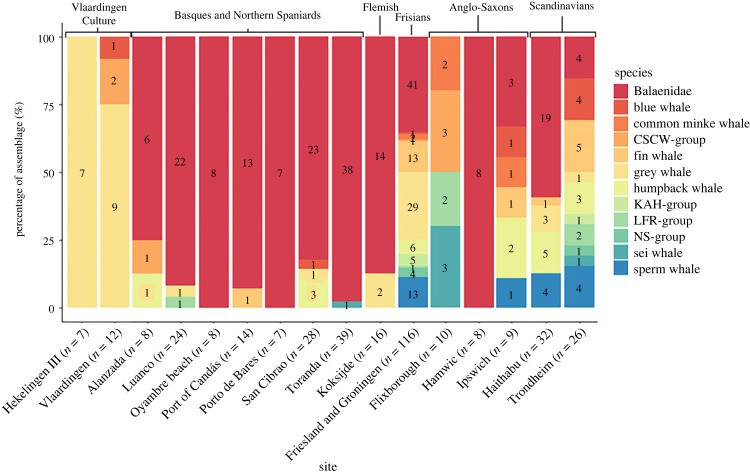
Percentages of ZooMS identifications for sites with many whale bones. CSCW-Group: common bottlenose/striped/common/white beaked dolphin; KAH-group: killer whale/Atlantic white-sided dolphin/harbour porpoise; LFR-group: long-finned pilot whale/false killer whale/Risso's dolphin; NS-group: northern bottlenose whale/Sowerby's beaked whale.

It is here argued that North Atlantic right whales and grey whales were often actively hunted by the Middle Ages at the latest, rather than exclusively harvested as stranded individuals. It can be difficult to differentiate these possibilities based on the archaeological record [15,60]. However, the ZooMS results (figure 2, electronic supplementary material, table S2 and figure S1) prove useful to assess this because active whaling is more likely if, as observed here, only particular taxa are well represented. Use of diverse taxa need not indicate the absence of whaling, but (within the limits of archaeological inference) specialized harvesting is a reasonable proxy for active hunting. Thus, based on the present dataset, it is also feasible to ask more specifically when and where in Atlantic Europe active whaling may have been practised in the past. With this objective, the percentage representation by identified whale taxon for well-represented sites or areas is provided in figure 4. These can be grouped according to seven relevant archaeological cultures. Six of these groups are mediaeval, and together formed a sequence of potential whale harvesting foci along the likely migration paths of *E. glacialis* and *E. robustus* in European waters, before their extirpation and extinction, respectively; based on the behaviour of *E. glacialis* from the western North Atlantic [61] and of Pacific grey whales [62], it is reasonable to hypothesize annual coastal migrations between southern calving and northern feeding grounds. The seventh archaeological culture is notable as a possibly much earlier (Neolithic) case of active whaling.

### 4.3. Mediaeval whaling

#### 4.3.1. Basques and northern Spaniards

Whale bones from seven sites from northern Spain were analysed as part of this study (A Lanzada (*ca* 400 BCE to 500 CE), Toranda (probably post 1290 CE), Port of Candás (probably post 1500 CE), San Cibrao (*ca* 100 BCE to 1800 CE), Oyambre beach (probably post 1612 CE), Porto de Bares (*ca* 1200–1800 CE) and Luanco (probably post 1622 CE)). Many of these specimens are not precisely dated, but the chronology of site occupation provides a date range or *terminus post quem*. The Basques are by far the most renowned early whalers based on written evidence [35], but historical whaling was also undertaken in other northern and northwestern regions of the Iberian Peninsula. The oldest documentary testimony of active whaling in northern Spain comes from a contract of Santoña (Cantabria) and the monastery of Nájera and is from the year 1190 CE [63], although some of our specimens pre-date this period. An aDNA study by Rey-Iglesia *et al*. [22] confirmed that whale bones recovered from early whaling harbours derive primarily from the North Atlantic right whale. The majority of our specimens also derive from the Balaenidae, most likely the North Atlantic right whale. We here identify one grey whale specimen from Spain (San Cibrao) and another from the French Basque region, although the latter is of a Roman date. These findings, in addition to the one specimen from Cudillero (previously thought to have derived from Luanco) identified by Rey-Iglesia *et al*. [22] and one from La Campa Torres, Gijon (dating to 400–200 BCE; [10]), suggest the grey whale was also present and occasionally used in northern Spain. Single specimens of other taxa may be indicative of incidental whaling or the exploitation of stranded individuals concurrent with active hunting.

#### 4.3.2. Normans

From the end of the eleventh century CE onwards, whaling in Normandy is mentioned in historical sources [33]. The North Atlantic right whale has been assumed the likely target [33]. The chronology of the specimens analysed here is varied, but one was identified as Balaenidae (seventh–eighth century CE) and four as grey whale (three from fifth century BCE Hérouvillette and one from a late second or early first century BCE context from Urville-Nacqueville).

#### 4.3.3. Flemish

Many historical sources, primarily concerned with the eleventh and twelfth centuries CE, suggest the existence of well-organized Flemish whaling (in what is now Belgium and northeast France) [34]. To infer whaling activities in Flanders, we analysed the site of Koksijde—Hof ter Hille (875–1125 CE) which provided 16 whale bone specimens. Fourteen of these were identified as Balaenidae (probably North Atlantic right whale), while the remaining two were identified as grey whale. Combined with the historical sources, this evidence suggests that the North Atlantic right whale was probably the main target of Flemish whalers, with the grey whale being targeted as well. Moreover, the sites of Raversijde (1270–1500 CE), De Haan—Kardinaal Mercierlaan (1000–1099 CE), Torhout (1000–1199 CE) and the castle site of Gravensteen in Ghent (1100–1199 CE) were also analysed, though these provided just one or a few of specimens each. These were again mainly identified as Balaenidae.

#### 4.3.4. Frisians

Limited historical evidence suggests the existence of active whaling activities undertaken by Frisians (in what is now The Netherlands and northwest Germany) [23]. There is not one Frisian site analysed here with large amounts of whale bone. Instead, various sites from the modern Dutch provinces of Friesland and Groningen have provided 116 whale bone specimens together. The diversity of species identified, and the scarce historical sources indicative of active whaling [23], suggest that opportunistic scavenging of stranded whales was the main procurement activity for the Frisians between approximately the eighth century BCE and the twelfth century CE. Many of the bones show signs of working; they were collected for the creation of a variety of artefacts. Nevertheless, a substantial portion of the bones derive from the Balaenidae (most likely North Atlantic right whale) and grey whale, implying the relative abundance of these species in the southern North Sea area.

#### 4.3.5. Anglo-Saxons

Little historical evidence exists for active whaling by the Anglo-Saxons in what is now England [36], but a number of archaeological sites have produced substantial numbers of whale bones. In order to infer possible active whaling activities during the Anglo-Saxon period, we assessed two proto-urban centres (Ipswich and Hamwic) and one high status rural settlement (Flixborough). Hamwic (with whale bones of seventh–twelfth century CE), as previously anticipated [64], exclusively provided Balaenidae specimens. Conversely, Ipswich (with specimens of seventh–twelfth century CE) produced a wide range of large whale taxa including Balaenidae, blue whale, fin whale, humpback whale, common minke whale and sperm whale. Flixborough (with relevant material of the seventh–eleventh century CE) differed again, yielding mainly common bottlenose dolphin (*Tursiops truncatus*), but also common minke whale and sei whale. The diversity of species at Ipswich may suggest opportunistic scavenging, although as observed for Haithabu and Trondheim (see below) this need not be so. The exclusive representation of Balaenidae at Hamwic may suggest active whaling, although the Anglo-Saxon inhabitants of this port might also have relied on trade with Norman whalers [64]. At Flixborough, the large number of common bottlenose dolphin specimens suggest a dedicated fishery targeting a local population, as previously argued by Dobney *et al*. [65]. No grey whale specimens derive from any of the three sites, though the contemporary Anglo-Saxon rural settlements of Botolphs and Carlton Colville have provided one grey whale specimen each, which could easily have been acquired through the opportunistic use of stranded individuals.

#### 4.3.6. Scandinavians

Whale bones from two relevant centres were analysed: Haithabu (in modern day Germany, but within the mediaeval kingdom of Denmark, dating *ca* 770–1066 CE) and Trondheim (whale bone specimens primarily dating to 1000–1500 CE). A variety of historical sources, the first as early as the ninth century CE, suggest that Scandinavians performed active whaling by the Viking Age (*ca* 800–1050 CE) at the latest. Several forms of whaling are described in mediaeval sources, including spearing (with or without poison), drives and trapping in bays or fjords [66]. The exploitation of stranded individuals, including division of carcasses, is also described in mediaeval Old Norse texts. The material from both Haithabu and Trondheim indicates a wide range of species. Thus, we observe that cultures involved in active whaling can produce taxonomically diverse assemblages. It seems unlikely either that all of the relevant specimens derived from active hunting, or that all were from strandings in the immediate hinterlands of the towns. Whale bone may instead have been brought to the proto-urban settlements from diverse locations for bone working. Indeed, the whale specimens from Trondheim all show signs of working, and have been fashioned into weaving swords, plaques or other objects. The excavations at Haithabu yielded a total of 55 gaming pieces made from whale bone [67]. Such whale bone pieces date to *ca* 550–1050 CE and are also quite frequently found in Sweden, signalling a large-scale distribution of North Atlantic whale products [24].

### 4.4. Neolithic whaling

#### 4.4.1. Neolithic Vlaardingen Culture

To explore the possibility of European whaling even earlier than the mediaeval period, we also considered material from the archaeological Vlaardingen Culture, where marine mammal bones have been recovered from several coastal sites [37]. The whale bones from the sites analysed here (Vlaardingen (*ca* 3500–2500 BCE) and Hekelingen III (*ca* 3000–2800 BCE) in particular, plus one whale bone specimen from Pompveld Altena (*ca* 3500–2500 BCE)) are predominantly of the grey whale. Additionally, the geographically closely positioned Neolithic site of Schipluiden (*ca* 3490–3380 BCE), which pre-dates the Vlaardingen Culture, has also provided one grey whale specimen. The very high proportion of grey whale findings from the Vlaardingen Culture sites suggests the species was abundant in the Rhine estuarine region and may imply that this particular taxon, which moves slowly in shallow coastal waters based on the behaviour of the Pacific grey whale [62], was actively hunted as early as the late forth to early third millennium BCE. The exploitation of marine resources was one of the main activities of the Vlaardingen Culture, as inferred from the high number of fish bones (including saltwater taxa) recovered from several sites, as well as the findings of fishing nets, an oak-dug canoe and paddles [37,68]. Neolithic seafarers are known to have successfully navigated the North Sea coast and crossed the English Channel [69]. Active whaling may have been practised concurrent with the harvesting of stranded whales. This case of probable active whaling in the Neolithic is a hitherto unparalleled (and still isolated) early manifestation of the practice in Continental Europe.

Overall, by combining previous historical and new ZooMS evidence it can be suggested that Balaenidae (most likely North Atlantic right) whales were the main target of mediaeval whaling activities, with grey whales targeted as well. The concentration of Balaenidae specimens identified at many of the mediaeval sites considered implies a long and persistent tradition of hunting along the Atlantic coast of Europe starting by or before the eleventh century and continuing up to the early twentieth century [52]. For the Neolithic period, the Vlaardingen Culture might have been specialized grey whale hunters in the Rhine estuary region. Further spatial and chronological detail regarding patterns of pre-industrial European whale utilization must await future research.

## 5. Conclusion

This study has highlighted the wealth of historical ecological data hidden in zooarchaeological and palaeontological archives of cetacean bone. Two baleen whale species that are no longer present in the eastern North Atlantic—Balaenidae (inferred to largely represent the North Atlantic right whale) and grey whale—were the taxa most identified in the subfossil record of large cetaceans. Early, especially mediaeval, whaling by multiple cultures along potential migration routes is hypothesized to have impacted the populations of both species, which must once have been frequently targeted. Though the eastern North Atlantic grey whale population may already have been declining due to climatic or ecological causes [51], it is suggested that a critical blow was dealt by mediaeval cultures undertaking whaling, contributing to the first extirpation of a cetacean species by anthropogenic activities. The North Atlantic right whale survived for a longer period of time in the eastern North Atlantic. It was still targeted by whalers there in the twentieth century [52], but had significantly declined in numbers by the eighteenth century [70], leading to the early modern expansion of Arctic and global whaling [71].

Both the grey whale and the North Atlantic right whale may have fallen victim to a perception of limitless natural abundance, due to ease of hunting and initially high numbers in coastal waters during the inception and early development of European whaling. After the pattern of targeting these once frequently encountered taxa became fixed in whaling practice, they were subsequently hunted to extirpation and extinction regardless of declining numbers. This phenomenon is not without parallels in the annals of human resource exploitation, be it of marine or terrestrial/avian species. The examples of the passenger pigeon [29], the European sea sturgeon [27] and the northern cod [26] were noted above, and it has previously been argued that the Atlantic walruses (*Odobenus rosmarus rosmarus*) of mediaeval Greenland suffered a similar fate [31,72]. In the case of the grey whale and North Atlantic right whale, the process unfolded over centuries and entailed comparatively long-lived migratory taxa, thus making it difficult for a downward trajectory to be fully recognized or successfully reversed. It remains to be established whether the frequent targeting of North Atlantic right whales and Atlantic grey whales implies that they were also once among the most abundant whales in European waters (rather than simply being the easiest to hunt). Based on the identification of subfossil specimens presented herein, this question of central importance to past (and potentially future) cetacean ecology must now be asked.

## Data Availability

Archaeological, context and ZooMS species identification data for the 719 specimens analysed are provided as electronic supplementary material, tables S1 and S2 [73]. The raw ZooMS spectra are published on the open-access Dryad Digital Repository: https://doi.org/10.5061/dryad.zgmsbcch7 [74].
